# Inter-individual Variation in Receptor Expression Influences MERS-CoV Infection and Immune Responses in Airway Epithelia

**DOI:** 10.3389/fpubh.2021.756049

**Published:** 2022-01-04

**Authors:** Kun Li, Christine Wohlford-Lenane, Jennifer A. Bartlett, Paul B. McCray

**Affiliations:** ^1^Department of Pediatrics, Carver College of Medicine, University of Iowa, Iowa City, IA, United States; ^2^Department of Microbiology and Immunology, Carver College of Medicine, University of Iowa, Iowa City, IA, United States

**Keywords:** DPP4, middle east respiratory disease (MERS), airway epithelia, individual variation, coronavirus, receptor, risk factors

## Abstract

Middle East respiratory syndrome coronavirus (MERS-CoV) causes respiratory infection in humans, with symptom severity that ranges from asymptomatic to severe pneumonia. Known risk factors for severe MERS include male sex, older age, and the presence of various comorbidities. MERS-CoV gains entry into cells by binding its receptor, dipeptidyl peptidase 4 (DPP4), on the surface of airway epithelia. We hypothesized that expression of this receptor might be an additional determinant of outcomes in different individuals during MERS-CoV infection. To learn more about the role of DPP4 in facilitating MERS-CoV infection and spread, we used ELISA and immunofluorescent staining to characterize DPP4 expression in well-differentiated primary human airway epithelia (HAE). We noted wide inter-individual variation in DPP4 abundance, varying by as much as 1000-fold between HAE donors. This variability appears to influence multiple aspects of MERS-CoV infection and pathogenesis, with greater DPP4 abundance correlating with early, robust virus replication and increased cell sloughing. We also observed increased induction of interferon and some interferon-stimulated genes in response to MERS-CoV infection in epithelia with the greatest DPP4 abundance. Overall, our results indicate that inter-individual differences in DPP4 abundance are one host factor contributing to MERS-CoV replication and host defense responses, and highlight how HAE may serve as a useful model for identifying risk factors associated with heightened susceptibility to serious respiratory pathogens.

## Introduction

In the past two decades, three highly pathogenic human coronaviruses have entered the world stage. The first, severe acute respiratory syndrome coronavirus (SARS-CoV), caused an epidemic in 29 countries in 2003 that resulted in more than 8,000 cases and nearly 800 deaths (World Health Organization, WHO). No cases of SARS-CoV have been identified since 2004. Middle East Respiratory Syndrome-CoV (MERS-CoV) is a novel β-coronavirus first isolated in Saudi Arabia in June of 2012 (1). Subsequently, MERS cases occurred in 27 countries and caused 858 deaths in 2,494 confirmed cases (World Health Organization, WHO). Unlike the short-lived SARS epidemic, MERS-CoV continues to circulate in the Arabian Peninsula (World Health Organization, WHO), probably because of continuous zoonotic transmission from local dromedary camels to humans (2). Most recently, the emergence of the novel coronavirus SARS-CoV-2 has led to the COVID-19 pandemic, which has sickened millions and continues to cause significant economic and societal upheaval worldwide.

The severity of MERS-CoV infection can vary widely between infected individuals. While many patients experience mild to moderate symptoms, a significant minority develop severe disease characterized by ARDS and multiorgan failure (3, 4). Severe MERS is associated with rapid virus replication, elevated pro-inflammatory cytokine/chemokine responses, and inflammatory cell infiltration [reviewed in (3, 5)]. It is not clear what factors determine such variability in outcomes. Male sex and immune compromise associated with older age or other co-morbid conditions are usually considered important risk factors. Another candidate host factor is the MERS-CoV receptor dipeptidyl peptidase 4 (DPP4, also known as CD26), which plays an important role in cell tropism, pathogenesis, and transmission (6).

DPP4 is differentially expressed in MERS-CoV susceptible species. In human lungs, DPP4 is expressed mainly in the lower respiratory tract in bronchiolar and alveolar type I and II epithelia, alveolar macrophages, vascular endothelia, and the pleural mesothelium (7, 8). In contrast, in dromedary camels DPP4 is expressed predominantly in the upper respiratory tract epithelium, with little alveolar expression (8). This expression pattern may contribute to the more efficient camel-to-camel or camel-to-human transmission as opposed to human-to-human spread of MERS-CoV, as well as to the milder disease observed in dromedary camels compared to humans.

Several animal models are available to study MERS-CoV infection and disease pathogenesis, including mice and non-human primates [reviewed in (4)]. However, due to species differences these models do not always faithfully recapitulate the spectrum of MERS pathophysiology observed in humans. To bridge the gap between the animal models and humans, *in vitro* infection models using human cell lines, primary cells, and *ex vivo* tissues are important resources. Airway epithelial cells are among the first targets of MERS-CoV infection. MERS-CoV was reported to infect non-ciliated cells in well-differentiated pseudostratified primary human airway epithelia, consistent with DPP4 expression in non-ciliated cells (6, 9). Later studies in *ex vivo* human lung tissue slices indicated that MERS-CoV also infects ciliated bronchial epithelia, non-ciliated cuboidal secretory cells, and alveolar type I and type II cells (10, 11). MERS-CoV infection caused apoptosis in both Calu-3 cells and epithelial cells in *ex vivo* human lung tissue slices (10–12). MERS-CoV infection also elicited antiviral host responses, with concomitant virus-encoded countermeasures (9, 10, 13, 14), indicating that the *ex vivo* airway epithelial cell models are robust representations of the *in vivo* infection.

In this study, we used well-differentiated human primary airway epithelia as a model to explore how DPP4 distribution and abundance relate to MERS-CoV tropism and infection. We found that DPP4 expression varies both between cell types and between individuals. In particular, we sought to better understand how variation in DPP4 at the level of the individual contributes to infection outcomes. Working from the hypothesis that greater levels of DPP4 would be associated with higher levels of infection and more severe pathology, we assessed DPP4 abundance in airway epithelial cultures from a large number of human lung donors, then asked whether inter-individual variability in DPP4 abundance correlates with viral replication and host antiviral responses. Our findings indicate that there is significant inter-individual variability in DPP4 abundance between donors, and that this variation in viral receptor abundance influences the overall level and severity of MERS-CoV infection in human airway epithelia.

## Materials and Methods

### Cell Culture

Primary airway epithelia (passage 0) were isolated from human donor bronchi and grown at air-liquid interface on collagen-coated, semi-permeable membranes with a 0.4 μm pore size (Costar Transwell; surface area 0.33 cm^2^; Corning) as reported previously (15). Human airway epithelial cultures were grown in DMEM/F12 with 2% Ultroser G (USG) media at 37°C with 5% CO_2_. All cell preparations used in this study were well-differentiated (>3 weeks old; resistance >1,000 Ohm x cm^2^). Using this culture method, we find that each Transwell filter typically supports approximately 300,000 cells. The study was approved by the Institutional Review Board at the University of Iowa. Calu-3 cells were first cultured in MEM supplemented with 20% FBS, 0.1 mM NEAA, 1 mM sodium pyruvate, 2 mM l-glutamine, 1% penicillin and streptomycin, and 0.15% NaHCO_3_ at 37° C with 5% CO_2._ When cells reached 80% confluence in submerged culture conditions, the Calu-3 cells were transferred to collagen-coated Transwell membranes as described (15) to generate air-liquid interface (ALI) cultures. Vero 81 cells were maintained in DMEM with 10% FBS and 1% penicillin and streptomycin at 37°C with 5% CO_2._

### Immunofluorescent Staining

Antibodies and reagents used for immunostaining are listed here: Anti-acetylated α-tubulin (Cell Signaling, cat#5335S), anti-DPP4 (R&D Systems, cat#AF1180), anti-MERS-CoV N protein (Sino Biological, cat#40068-MM10), anti-cleaved caspase-3 (Cell Signaling, cat#9664), and Alexa Fluor 647 phalloidin (ThermoFisher Scientific, cat#A22287). Methods for immunolocalization of DPP4 in human airway epithelia were adapted from previous studies (7). Briefly, airway epithelia or cytospun BAL samples were fixed in 4% paraformaldehyde and permeabilized with 0.2 % Triton X-100. Following incubation with SuperBlock™ buffer, primary antibodies were applied to the fixed cells on slides, or to the apical and basolateral cell surfaces of epithelial sheets overnight, followed by washing with SuperBlock™ buffer three times. After a 1 h incubation with the appropriate Alexa Fluor secondary antibodies, followed by more washes, coverslips were mounted using Vectashield antifade mounting medium with DAPI (Vector Laboratories). Photomicrographs were taken using a Leica TCS SPE confocal microscope. The number and types of infected cells were counted manually using ImageJ.

### MERS-CoV Infections

The EMC/2012 strain of MERS-CoV (passage 8, designated MERS-CoV) was provided by Dr. Bart Haagmans and Ron Fouchier (Erasmus Medical Center). To infect airway epithelia, cultures were first rinsed apically with 200 μl sterile PBS to removed shed DPP4 from the cell surface. Viral inoculum was then applied to the apical surface of cells (in a volume of 50 μl per Transwell) for 1 h at 37°C, 5% CO_2_. Following this incubation period, virus was removed, and cells were rinsed three times with 200 μl PBS to remove unbound virus. Infected cultures were maintained at 37°C, 5% CO_2_. At the indicated time points, apical airway surface liquid (ASL) was collected by washing the surface with 60 μl sterile PBS and stored at −80°C for later titering or measurement of cellular genomic DNA. To remove mucus, 50 μl of 10 mM DTT in PBS containing Ca^2+^ and Mg^2+^ (Gibco, cat#14040133) was added to the apical surface of airway epithelia and incubated at 37°C, 5% CO_2_ for 10 min. After washing with PBS three times, treated cells were incubated at 37°C, 5% CO_2_ for 2 h to allow tight junction recovery before infection with MERS-CoV.

### Measurement of Viral Replication

Virus was propagated and titered by plaque assay as described in (16). Briefly, thawed apical wash samples were serially diluted in serum-free DMEM and applied to the surface of Vero 81 cells. Cells were incubated with virus at 37°C, 5% CO_2_, with gentle rocking. After 1 h, virus was removed and replaced by a 1% agarose overlay. Cells were incubated for 3 days at 37°C, 5% CO_2._ To visualize plaques, cells were fixed with 25% formaldehyde and stained with 0.1% crystal violet. Titers are expressed as plaque-forming units (PFU) per ml. Plaque assays were performed at the Biosafety Level 3 (BSL3) facility at the University of Iowa.

### DPP4 ELISA and Flow Cytometry

To quantify cellular DPP4 abundance, human airway epithelial cultures and Calu-3 cells were rinsed apically with sterile PBS, then lysates were prepared by resuspending cells in buffer containing 1% NP-40 (100 μl lysis buffer per Transwell culture). Lysates were spun in a tabletop centrifuge for 5 min at 1000 rpm to pellet cell debris, and DPP4 protein abundance in the supernatants was measured using the human DPP4IV/CD26 Duoset ELISA kit (R&D Systems, cat#DY1180) following the manufacturer's protocol.

To identify the cell types expressing DPP4, airway epithelia were detached from Transwell inserts by incubation with Accumax (Innovative Cell Technologies), then resuspended and washed in ice cold wash buffer (PBS with 2% FBS). Next, cells were fixed and permeabilized using a fixation/permeabilization solution from the Cytofix/Cytoperm kit (BD Bioscience, cat#554714). After blocking with SuperBlock™ buffer (ThermoFisher Scientific, cat#37515), cells were incubated with anti-DPP4 antibody (1:50) (R and D Systems, cat#AF1180) for 1 h followed by Alexa Fluor488 (ThermoFisher Scientific). Cells were analyzed using a BD Accuri C6 flow cytometer (BD Biosciences).

### Measurement of Cellular Genomic DNA in Airway Surface Liquid

To confirm sloughing of MERS-CoV infected cells, the apical surface of infected or mock infected airway epithelial cultures were rinsed with 100 μl PBS at the indicated time points. After three freeze-thaw cycles, genomic DNA in the rinses was quantified using the Qubit dsDNA High Sensitivity Assay Kit (Invitrogen, cat#Q32854), according to the manufacturer's instructions.

### Quantitative RT-PCR to Measure MERS-CoV Viral RNA and Host Defense Genes

Total RNA was isolated from infected airway epithelia using TRIzol™ (Life Technologies) and the Direct-zol RNA MiniPrep kit (Zymo Research, Irvine, CA). A DNase treatment step was included. Total RNA (200 ng) was used as a template for first strand cDNA synthesis with the High-Capacity cDNA Reverse Transcription Kit (Applied Biosystems, cat#4368814). The resulting cDNA was subjected to amplification of selected genes by quantitative real-time PCR (qRT-PCR) using Power SYBR Green PCR Master Mix (Applied Biosystems, cat#4367695). Samples were run in duplicate and averaged. Averages were then used to calculate the relative abundance of transcripts normalized to HPRT and presented as 2^−Δ*CT*^. The sequences for all primers used are listed in Table 1.

**Table 1:** Primer Sets for qRT-PCR.

**Genes**		**Sequence (5^′^-3^′^)**
MERS-CoV ORF1a	Forward	CCACTACTCCCATTTCGTCAG
	Reverse	CAGTATGTGTAGTGCGCATATAAGCA
HPRT	Forward	AGG ATT TGG AAA GGG TGT TTA TTC
	Reverse	CAG AGG GCT ACA ATG TGA TGG
IFN-α	Forward	GAC TCC ATC TTG GCT GTG A
	Reverse	TGA TTT CTG CTC TGA CAA CCT
IFN-β	Forward	GCC GCA TTG ACC ATC T
	Reverse	CAC AGT GAC TGT ACT CCT
IFN-λ	Forward	GAA GCA GTT GCG ATT TAG CC
	Reverse	GAA GCT CGC TAG CTC CTG TG
ISG15	Forward	CAT GGG CTG GGA CCT GAC G
	Reverse	CGC CAA TCT TCT GGG TGA TCT G
IFITM3	Forward	TGT CCA AAC CTT CTT CTC TCC
	Reverse	CGT CGC CAA CCA TCT TCC
OAS1	Forward	CAA GCT CAA GAG CCT CAT CC
	Reverse	TGG GCT GTG TTG AAA TGT GT
IP-10/CXCL10	Forward	CTG ACT CTA AGT GGC ATT
	Reverse	TGA TGG CCT TCG ATT CTG
CXCL2	Forward	GGG CAG AAA GCT TGT CTC AA
	Reverse	GCT TCC TCC TTC CTT CTG GT
MCP-1/CCL2	Forward	GCA ATC AAT GCC CCA GTC A
	Reverse	TGC TGC TGG TGA TTC TTC TAT AGC T
IL-1α	Forward	TGG TAG TAG CAA CCA ACG GGA
	Reverse	ACT TTG ATT GAG GGC GTC ATT C
IL-1β	Forward	CCT GTC CTG CGT GTT GAA AGA
	Reverse	GGG AAC TGG GCA GAC TCA AA
IL-6	Forward	GGT ACA TCC TCG ACG GCA TCT
	Reverse	GTG CCT CTT TGC TGC TTT CAC
IL-8	Forward	AGC TGG CCG TGG CTC TCT
	Reverse	CTG ACA TCT AAG TTC TTT AGC ACT CCT T
IL-10	Forward	GCT GGA GGA CTT TAA GGG TTA CCT
	Reverse	CTT GAT GTC TGG GTC TTG GTT CT

### Statistical Analysis

Statistical tests were performed using GraphPad Prism 7. Unpaired, 2-tailed Student's *t*-tests, Mann Whitney test, or Wilcoxon matched-pairs signed rank test were used to analyze differences in mean values between groups, as indicated. Simple linear regression was used to test for correlations between DPP4 protein abundance and other variables. For datasets representing timecourse data, one-way ANOVA followed by Dunnett's or Sidak's multiple comparisons tests were performed to test for statistically significant differences from baseline values. All results are expressed as mean ± SE, and *P* < 0.05 were considered significant.

## Results

### MERS-CoV Infection Features Dynamic Changes in Cell-Specific Tropism

**Figure 1:**
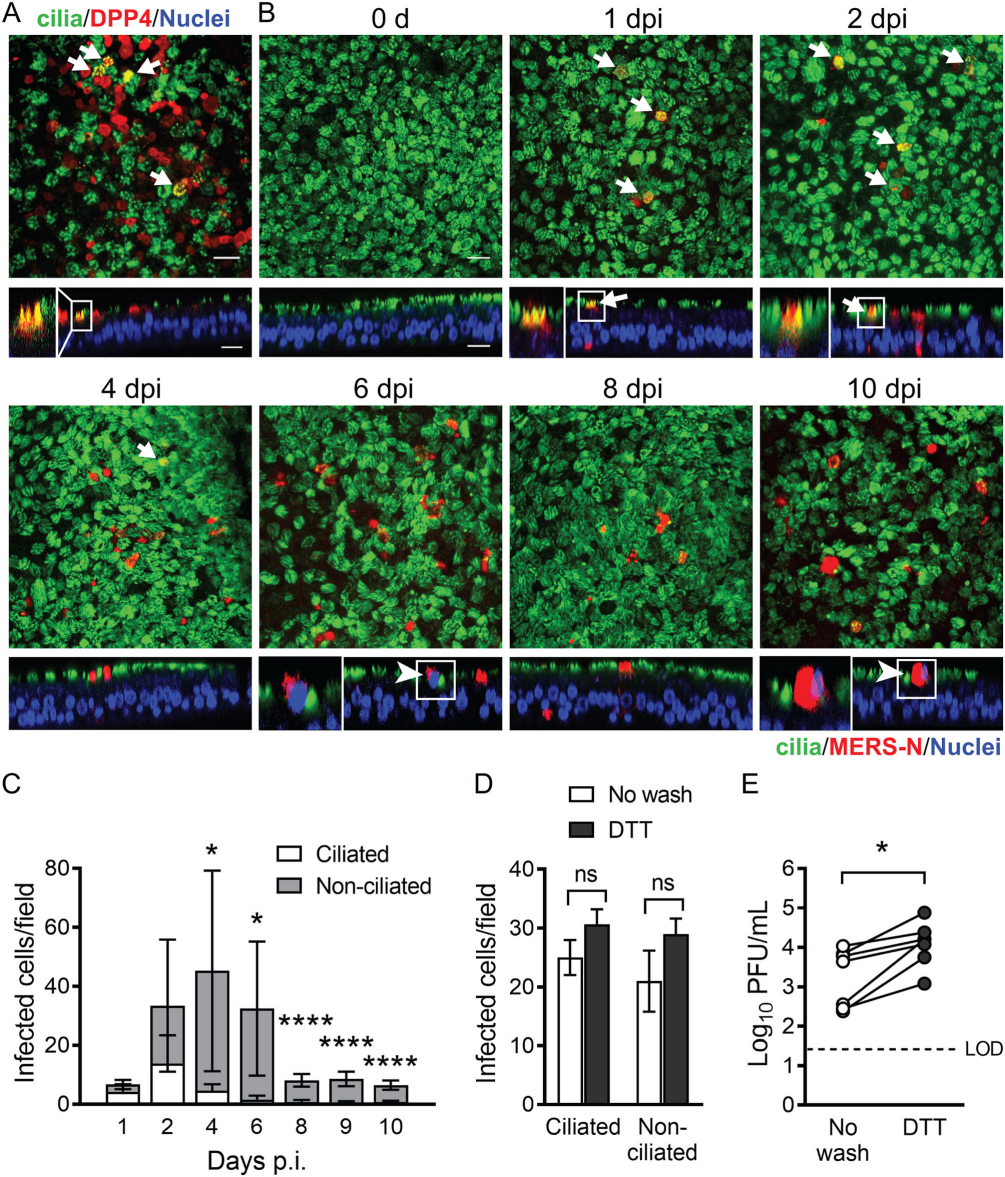
MERS-CoV infection and spread in human airway epithelia. **(A)** DPP4 distribution in HAE demonstrated by immunofluorescence (DPP4, red; nuclei, blue). Ciliated cells are indicated by positivity for acetylated tubulin (green). DPP4 is expressed on the apical surface of nonciliated and occasional ciliated cells (yellow; white arrows, inset). Scale bar = 20 μm. **(B)** HAE were infected with MERS-CoV (MOI = 0.1) from the apical surface. At the indicated time points, cells were fixed and immunostained for viral antigen (N protein, red) and cilia (acetylated α-tubulin, green), followed by DAPI staining of nuclei (blue). White arrows indicate infected ciliated cells. Arrowheads denote infected cells on top of apical surface of the epithelium. Scale bar = 20 μm. **(C)** The number of infected ciliated and non-ciliated cells per 40X field at the indicated time points post infection were determined by visual counting (3 fields/HAE donor x 3 donors). Data represent mean ± standard deviation. Asterisks denote statistically significant differences between infected ciliated vs. non-ciliated cells at each time point, as determined by performing multiple unpaired *t*-tests with Welch correction. *P < 0.05; ****P < 0.0001. **(D)** Mucus was removed from the apical surface of HAE cultures by incubation with 10 mM DTT followed by PBS rinses, as described in Methods. HAE cultures with or without mucus removed were then infected apically with MERS-CoV (MOI = 0.1). The total numbers of infected ciliated and non-ciliated cells were counted at 1 day post infection for the mucus replete (No wash) and mucus-depleted (DTT) conditions (4 fields/HAE donor x 3 donors). Mann-Whitney test was performed to test for significant differences between the No wash and DTT conditions for each cell type (ns = not significant). **(E)** HAE (*n* = 7 donors) were infected with MERS-CoV (MOI = 0.1), with and without mucus removal, and plaque assay was used to measure release of viral progeny at 1 day post infection. Each data point represents results from a single HAE donor. The Wilcoxon matched-pairs signed rank test was used to test for significant differences between paired titer data (No wash vs. DTT) for each HAE donor *P < 0.05.

Earlier studies suggested that MERS-CoV infection is mostly restricted to non-ciliated airway epithelial cells (6, 9). To investigate the cell-type distribution of the MERS-CoV receptor in airway epithelia, we used immunocytochemistry to localize DPP4 protein expression in well-differentiated cultures of primary human airway epithelia (HAE) derived from human lung donors (Figure 1A). In agreement with previous reports that DPP4 is expressed only on the apical surface of non-ciliated airway epithelial cells (6), we observed the protein predominantly on the apical surface of non-ciliated cells. We also occasionally observed DPP4 on the apical surface of some ciliated cells (arrows and inset, Figure 1A). This finding is consistent with previous studies of DPP4 protein expression in human tissue samples (7). It is also supported by single cell RNA sequencing studies using lung tissue samples and primary airway epithelia, which indicate that while the predominant cell types expressing DPP4 in the respiratory tract are alveolar type II cells and secretory cell types, DPP4 transcripts can also be detected in a subset of the ciliated cell population (17–20).

Noting these occasional DPP4-positive ciliated cells, we wondered whether MERS-CoV might also have tropism for a subset of ciliated cells in addition to non-ciliated cell types in this culture model of the surface airway epithelium. To address this question, we infected HAE apically at 0.1 MOI and monitored MERS-CoV spread over a 10-day timecourse. As shown in Figure 1B, the overall number of infected cells increased between 1 and 4 days post infection and then declined after day 6. During the early stages of the timecourse (1–2 days post infection), we observed infection in both ciliated and non-ciliated cells (Figures 1B,C). After the first 1–2 days, the incidence of infected ciliated cells began to decline, coinciding with the decline in the overall number of infected cells after day 6. This may reflect release of infected cells onto the apical surface, as we observed nuclei of infected cells at the apical surface [(Figure 1B, 6 and 10 days post infection (arrowheads and insets)].

Secretory non-ciliated cells produce mucins that entrap inhaled pathogens (21, 22), and the ciliated cells interact with the mucus to sweep pathogens and other particulates from the airways (23). Because secreted mucus might hinder the MERS-CoV infection cycle, we removed it by rinsing the cell surface with the reducing agent DTT prior to virus inoculation (24). The total number of infected cells modestly increased after mucus removal (Figure 1D). Following mucus removal, the apical release of progeny virus increased significantly at 1 day post infection (Figure 1E). Together, these findings indicate mucus may interfere with virus entry and/or release.

### DPP4 Expression Varies Greatly Among Airway Epithelia From Different Donors

**Figure 2:**
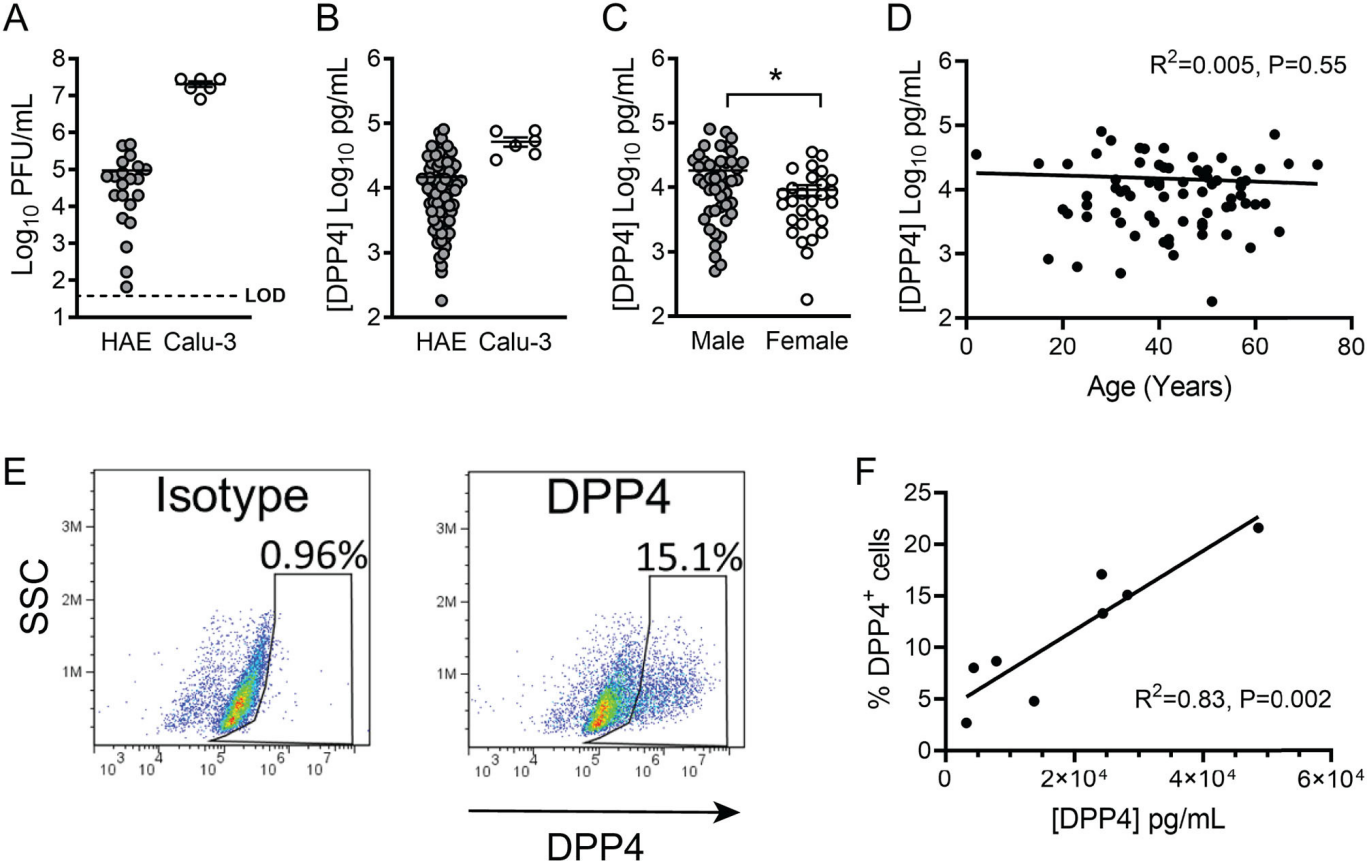
DPP4 expression in human airway epithelia. **(A)** Air-liquid interface cultures of primary human airway epithelia (HAE) or Calu-3 cells were infected apically with MERS-CoV at an MOI of 0.1, and viral release into the apical compartment at 1 day post infection was quantified by plaque assay. (HAE, *n* = 21; Calu-3, *n* = 6) Each data point represents results from a single HAE donor. LOD denotes limit of detection. In all graphs, data represent the mean ± SE. **(B)** Cell-associated DPP4 abundance was determined by ELISA for HAE (*n* = 74) or Calu-3 cells (*n* = 6). For each donor, the result represents the DPP4 protein concentration in a lysate prepared from a single Transwell filter (see Methods for additional details). **(C)** Measured DPP4 abundances were compared according to sex (male, *n* = 46; female, *n* = 28) and data were tested for significant differences using unpaired 2-tailed *t*-test. *P* < 0.05. **(D)** DPP4 abundance and HAE donor age were correlated using linear regression. **(E)** Representative plot showing the percentage of DPP4^+^ cells per Transwell epithelial sheet, as measured by flow cytometry. **(F)** Correlation between cellular DPP4 abundance and percentage of DPP4^+^ cells (assessed by flow cytometry) in HAE cultures (*n* = 8).

Interestingly, we noticed that when we infected HAE with MERS-CoV, the titer of apically released viral progeny at 1 day post infection was quite variable among cells derived from different donors (Figure 2A). In contrast, such variation was not observed in infected air-liquid interface (ALI) cultured Calu-3 airway epithelial cells (Figure 2A). We hypothesized that this variability in MERS-CoV infection in primary airway cells might reflect lung donor variability in DPP4 expression. To explore this, we used a human DPP4-specific ELISA to directly measure DPP4 abundance in cell lysates from HAE representing 74 individual donors (Figure 2B). Prior to preparing cell lysates, HAE cultures were washed extensively to remove shed DPP4, ensuring that measurements represent membrane-bound DPP4. We found that DPP4 abundance varied markedly among HAE donors, ranging from ~10^2^ to ~10^5^ pg/ml [1.48 x 10^4^ ± 1.9 x 10^3^ pg/ml (mean ± SE)], while DPP4 abundance was consistently high in ALI cultures of Calu-3 cells (5.2 x 10^4^ ± 8.6 x 10^3^ pg/ml). According to WHO data, approximately two thirds of MERS cases were diagnosed in males and the median age of subjects was ~50 years. Therefore, we examined DPP4 protein abundance in this dataset when stratified by donor sex (Figure 2C) and age (Figure 2D). While there was not a significant relationship between HAE donor age and DPP4 abundance, we did observe significantly greater DPP4 abundance in epithelia from male donors compared to cells from female donors, suggesting that sex differences in DPP4 protein expression may contribute to the greater susceptibility of males to MERS-CoV infection. To further characterize DPP4 expression and distribution in cultured airway epithelia, we evaluated the percentages of DPP4-expressing cells by flow cytometry (Figure 2E). We found that the percentage of cells expressing DPP4 correlated with the DPP4 abundance of each epithelium as measured by ELISA (Figure 2F).

### Cellular DPP4 Abundance Influences MERS-CoV Replication Efficiency

**Figure 3:**
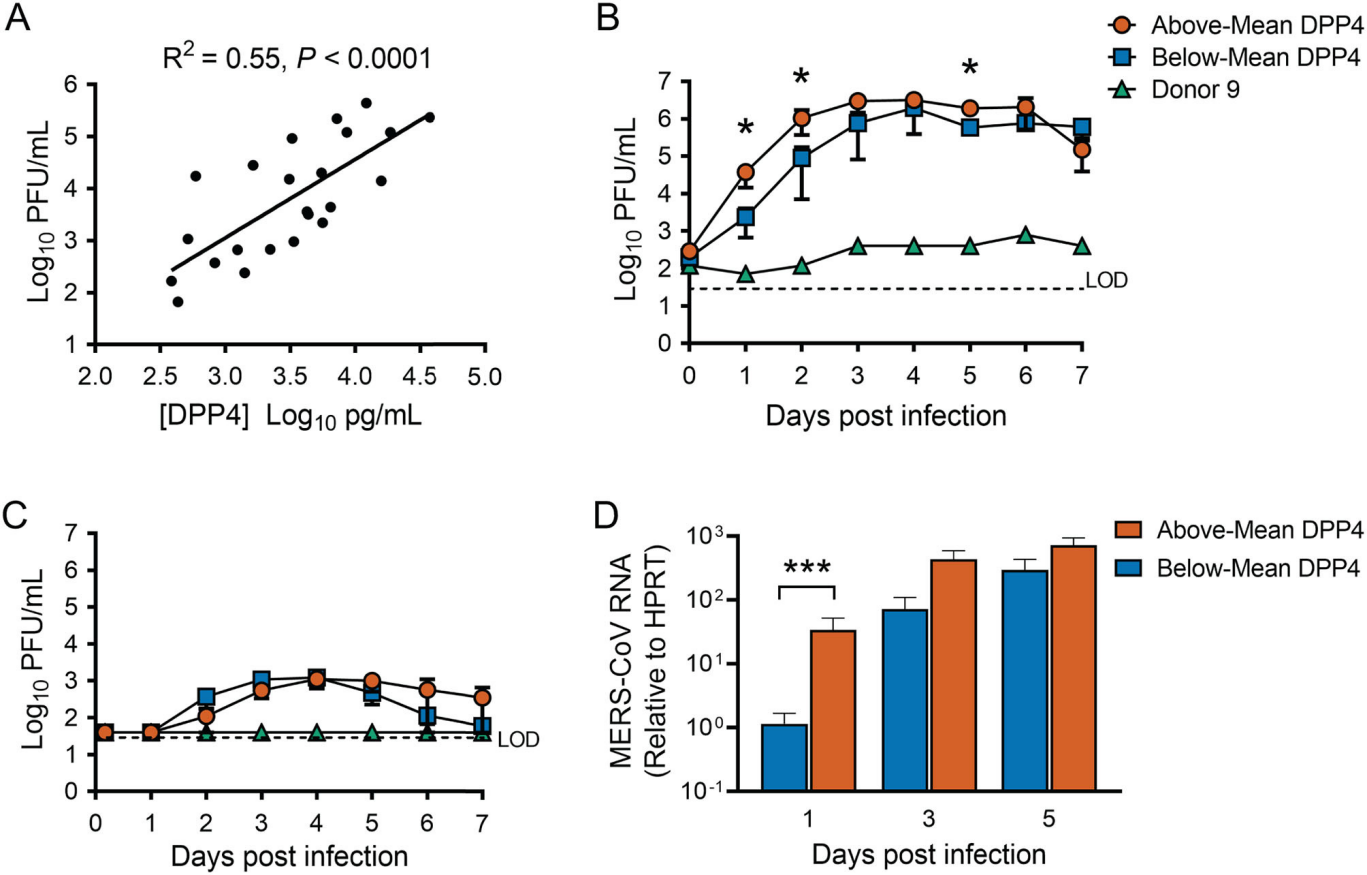
Cellular DPP4 abundance influences MERS-CoV replication in human airway epithelia. **(A)** HAE (*n* = 23) were infected apically with MERS-CoV at an MOI of 0.1, and apical release of progeny virus at 1 day post infection was quantified by plaque assay. Linear regression was performed to assess the relationship between progeny virus titers and cellular DPP4 abundance. **(B)** HAE were infected apically with MERS-CoV (MOI = 0.1), and viral growth was monitored over a 7-day timecourse by measuring release of progeny virus from the apical **(B)** or the basolateral compartment **(C)**. Above-Mean DPP4 group, *n* = 4; Below-Mean DPP4 group, *n* = 4. LOD denotes limit of detection. **(D)** Viral replication was also monitored by QPCR to detect MERS-CoV genomic RNA levels in infected cells. In **(B–D)**, data were tested for significant differences between the Above-Mean and Below-Mean groups at each timepoint by performing unpaired 2-tailed *t*-tests on log transformed data. *P < 0.05, ***P < 0.001. In all graphs, data are presented as mean ± SE.

We noted that the titers of released virus at 1 day post infection correlated positively with cellular DPP4 abundance (Figure 3A), suggesting that cellular DPP4 abundance at least partly determines individual susceptibility to MERS-CoV. To explore the relationship between DPP4 abundance and MERS-CoV infection, we infected HAE *via* apical inoculation with MERS-CoV (MOI = 0.1) and measured virion production over a 7-day timecourse. For this experiment, 9 HAE donors were selected from our larger dataset of 74 donors (Figure 2B). The donor specimens were selected to represent the range of DPP4 concentrations that were observed across the dataset and were divided into two groups: an Above-Mean group (Donors 1–4), cells with DPP4 abundance above the mean in the large dataset, and the Below-Mean group (Donors 5–8), epithelia with DPP4 abundance that was below the average. Cells from one additional HAE donor, that exhibited particularly low DPP4 abundance (Donor 9), was also selected for this study. Measured DPP4 abundances for the individual HAE donors used in this experiment are summarized in Table 2.

**Table 2:** Cellular DPP4 abundance in HAE donor specimens used for MERS-CoV infection studies.

	**HAE donor**	**DPP4 abundance (pg/ml)**
Above-Mean DPP4 Group	Donor 1	21,702
	Donor 2	35,279
	Donor 3	25,225
	Donor 4	19,828
Below-Mean DPP4 Group	Donor 5	10,813
	Donor 6	13,427
	Donor 7	5,431
	Donor 8	2,221
Low DPP4	Donor 9	629

We found that MERS-CoV replicated efficiently in all epithelia from both the Above- and Below-Mean groups, reaching peak titers of ~10^6^ PFU/ml in apical rinse material collected around 3 to 6 days post infection (Figure 3B) and up to ~10^3^ PFU/ml in the basolateral medium at 3–4 days post infection (Figure 3C). The ratio of peak virion release from the apical vs. the basolateral surface was at least ~100, indicating preferential release from the apical surface. Overall, progeny virus titers plateaued at 3 or 4 days post infection and remained stable for the remainder of the experiment, with the titers for the Above-Mean DPP4 group exhibiting a slight decline between 6 and 7 days post infection (Figure 3B). Notably, the virus replicated poorly in epithelia from the donor with very low DPP4 abundance (Donor 9). We also quantified the abundance of viral RNA in infected cells by qRT-PCR (Figure 3D). In keeping with the viral titer data, MERS-CoV RNA steadily increased throughout the timecourse in both groups and peaked at around 3 to 5 days post infection. Interestingly, HAE donors in the Above-Mean DPP4 group reached peak virion production ~1 day earlier than donors in the Below-Mean group (Figure 3B), and exhibited significantly greater viral titers and RNA levels at 1 day post infection (Figure 3D). These data point to greater viral loads in the Above-Mean DPP4 group at the early time points and suggest that the greater DPP4 expression in this group may have contributed to more efficient viral replication in the early phase of the infection.

### DPP4 Abundance Influences Sloughing of Airway Epithelia During MERS-CoV Infection

**Figure 4:**
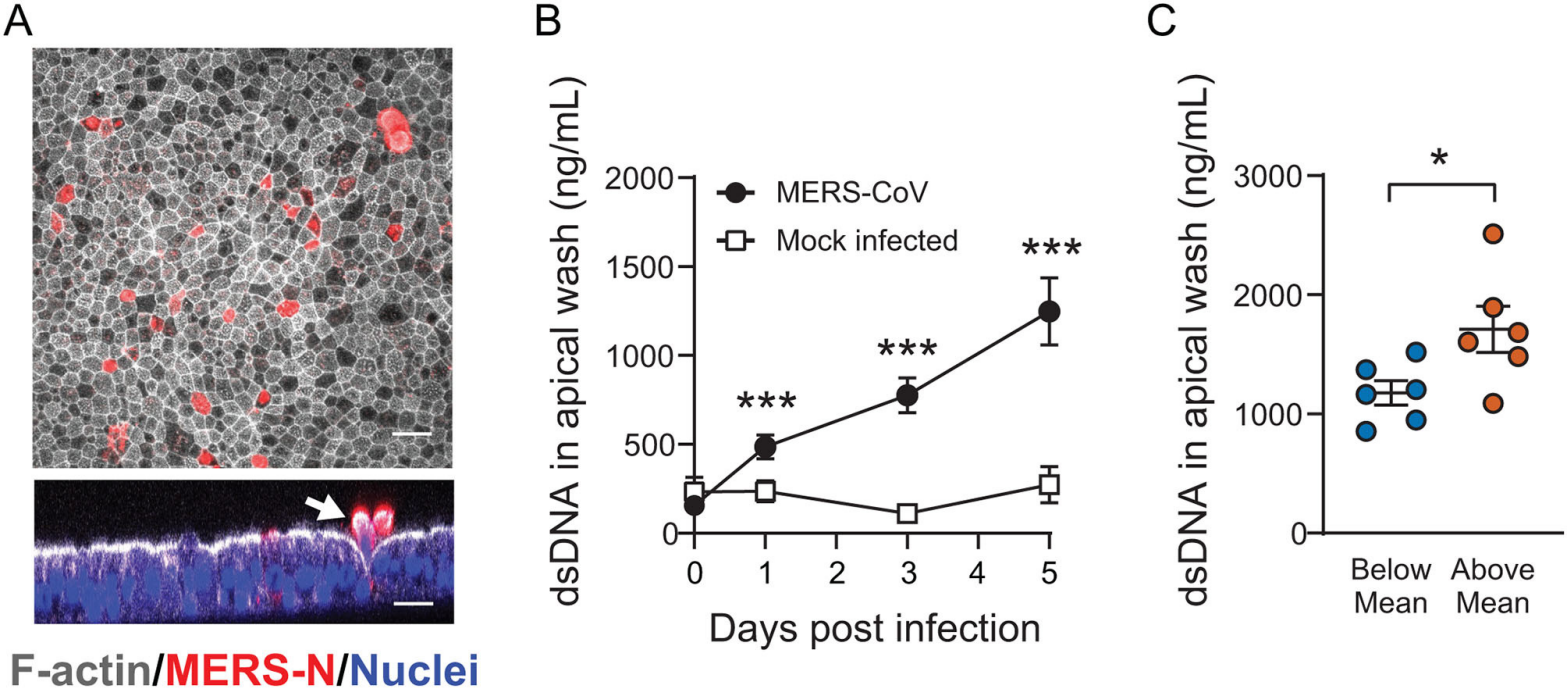
The impact of cellular DPP4 abundance on MERS-CoV induced airway epithelial sloughing. **(A)** Following MERS-CoV infection (MOI = 0.1), HAE were fixed and stained with anti-MERS N protein antibody, phalloidin, and DAPI to identify infected cells (red), filamentous actin (white), and nuclei (blue), respectively. In the cross sectional image, infected cells are seen being expelled from the epithelial surface (white) at 4 days post infection (arrow). Scale bar = 20 μm. **(B)** To test for shed cells, the apical compartments of MERS-CoV infected cultures (*n* = 10) or mock infected cultures (*n* = 3) were sampled by rinsing with PBS and the abundance of cellular genomic DNA was measured in the collected material at the indicated time points. For both groups, data were tested for significant increases in shed DNA relative to baseline (Day 0) by repeated measures one-way ANOVA followed by Tukey's multiple comparisons test ***P_*Adj*_ < 0.001. **(C)** The abundance of cellular genomic DNA in apical washes was compared for HAE with Above-Mean or Below-Mean DPP4 expression at 5 days post infection (*n* = 6 for both groups). The groups were tested for significant differences by unpaired 2-tailed *t*-test *P < 0.05. In **(B,C)**, data are plotted as mean ± SE.

In our timecourse studies, we observed that the overall numbers of infected epithelial cells declined over time, suggesting shedding of infected and/or apoptotic cells (Figures 1B,C). Using immunofluorescent staining, we confirmed that MERS-CoV infected cultures exhibited evidence of epithelial cell sloughing (Figure 4A). To characterize shed cells, we washed the apical surface of infected (or mock infected; time = 0) HAE at 1, 3, and 5 days post infection, and quantified the abundance of cellular genomic DNA in the washes, a correlate of the number of sloughed cells. MERS-CoV infection triggered a progressive loss of cells over time (Figure 4B), which was significantly more pronounced in donors whose DPP4 abundance was above the mean (Figure 4C).

### Induction of Host Innate Immune Genes During MERS-CoV Infection

**Figure 5:**
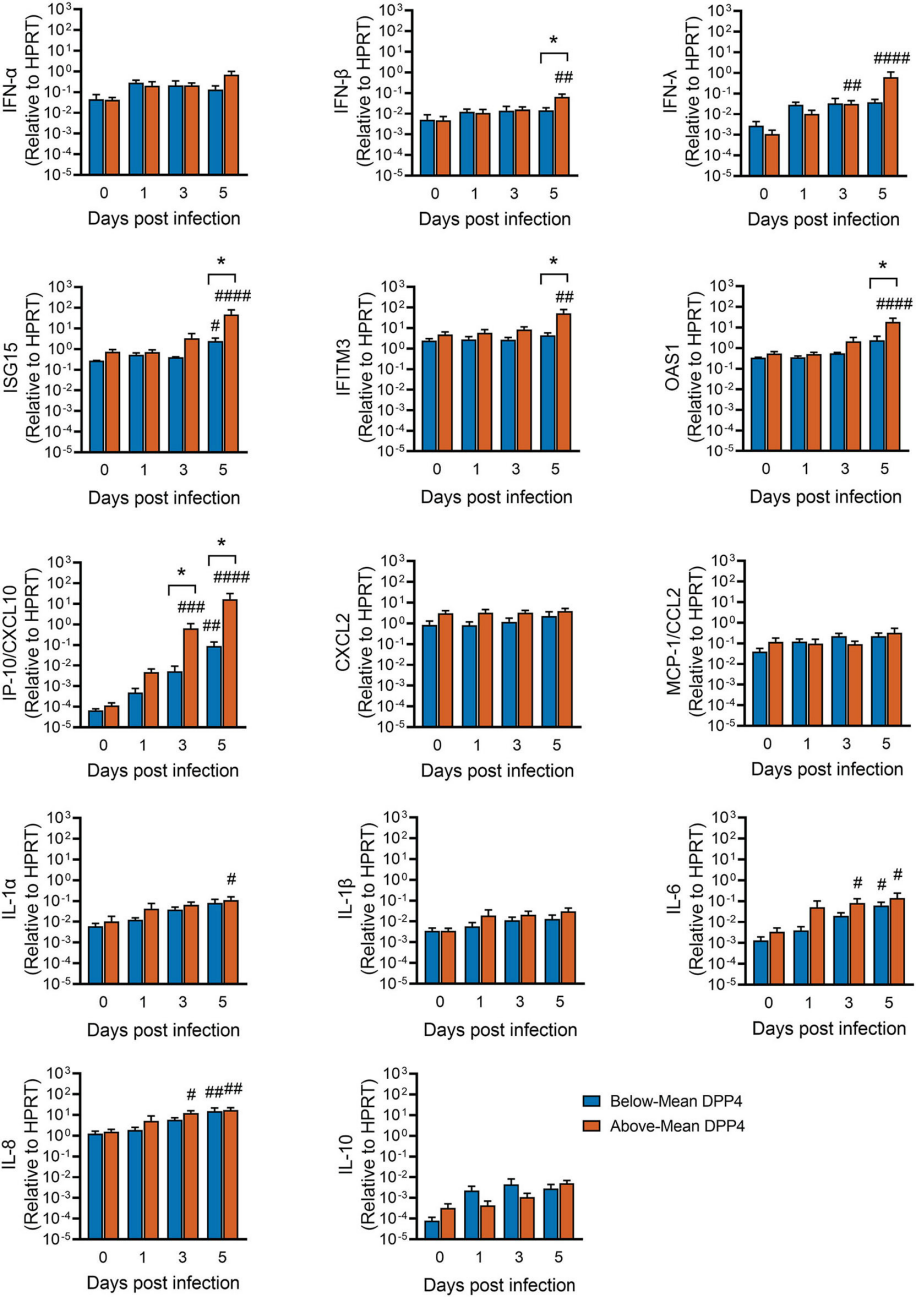
Innate immune responses following MERS-CoV infection and relationship to DPP4 abundance. HAE were infected apically with MERS-CoV (MOI = 0.1) and total RNA isolated at the indicated time points for qRT-PCR analysis. For each gene, mRNA abundance was normalized to that for HPRT. Above-Mean DPP4 group, *n* = 4; Below-Mean DPP4 group, *n* = 4. Data represent mean ± SE. One-way ANOVA, followed by Sidak's multiple comparisons test, was used to test log transformed data for significant differences relative to the corresponding uninfected control cells (Day 0) within each group. ^#^*P*_*adj*_ < 0.05, ^*##*^*P*_*adj*_ < 0.01, ^*###*^*P*_*adj*_ < 0.001, ^*####*^*P*_*adj*_ < 0.0001. To test for significant differences between the Above- and Below-Mean groups at each time point post infection, log transformed data were tested by unpaired 2-tailed *t*-tests *P < 0.05.

Given these DPP4-dependent differences in viral replication kinetics and cell death, we hypothesized that host defense responses might also differ according to DPP4 abundance. The median incubation period for MERS-CoV is estimated to be in the range of 5–7 days (25). To learn more about how the virus interacts with the host immune response in this critical early phase of infection, we used quantitative RT-PCR to measure expression of innate immune genes involved in antiviral defense in our MERS-CoV timecourse dataset (Figure 3). MERS-CoV has evolved several mechanisms to evade host immune responses, including inhibiting the induction of type I IFN (IFN-α, IFN-β) (9, 10, 13, 14). Consistent with this, we observed that the type I interferon response was relatively delayed in MERS-CoV infected epithelia, with significant induction of IFN-β gene transcripts seen only at day 5 post infection and only in the Above-Mean DPP4 group (Figure 5). Induction of type III interferon (IFN-λ) mRNA was similarly delayed, with significant increases seen in the Above-Mean DPP4 group at 3 and 5 days post infection.

Mirroring this lag in induction of the interferon response, we observed that transcripts for many host defense genes were significantly upregulated from baseline levels only relatively late in the timecourse (3–5 days post infection) (Figure 5). This trend was observed for several interferon-stimulated genes and pro-inflammatory cytokines, including interferon-stimulated gene 15 (ISG15), interferon induced transmembrane protein 3 (IFITM3), 2′-5′-oligoadenylate synthetase 1 (OAS1), C-X-C motif chemokine ligand 10 (CXCL10, also known as IP-10), interleukin-1α (IL-1α), interleukin 6 (IL-6), and interleukin 8 (IL-8). It was also at this late timepoint that differences became apparent between the Above- and Below-Mean DPP4 groups, with the Above-Mean DPP4 donors showing greater induction of IFN-β, ISG15, IFITM3, and OAS1. Notably, MERS-CoV infection markedly increased IP-10/CXCL10 mRNA in well-differentiated airway epithelia. This upregulation was particularly pronounced in donors from the Above-Mean DPP4 group, who exhibited a >100-fold increase in IP-10/CXCL10 transcripts relative to donors in the Below-Mean DPP4 group.

## Discussion

A main objective of this study was to examine the relationship between individual variation in the MERS-CoV receptor, DPP4, and variability in MERS-CoV susceptibility and disease outcomes. Using primary airway epithelia from human donors as a model, we found that DPP4 abundance correlated positively with infection and viral replication, and appeared to influence pathogenesis as assessed by cell sloughing/disruption of the epithelium. We also detected a relationship between DPP4 abundance and progression of the host innate immune response, with high-DPP4 airway epithelial donors exhibiting relatively greater induction of several antiviral genes in response to MERS-CoV infection. Overall, our findings support the idea that individual variation in DPP4 expression–in addition to sex and age-should be considered a determinant of MERS-CoV pathogenesis and transmission in humans.

We found that DPP4 abundance is subject to significant inter-individual variability, with DPP4 varying as much as 1000-fold between individuals (Figure 2). DPP4 expression is dynamically regulated by a number of stimuli, and it has been reported that disease states including inflammation, chronic lung disease, cancer, obesity, and diabetes, can increase DPP4 expression in the lungs and other organs (7, 26–28). This suggests that upregulation of DPP4 may be one mechanism by which underlying co-morbidities increase the risk of MERS-CoV infection. Interestingly, we observed one HAE donor (Donor 9) whose measured DPP4 abundance was particularly low and which did not seem to support MERS-CoV replication (Figure 3). Based on this observation, we speculate that in some individuals, DPP4 expression may actually be low enough-due to genetics or other factors-to reduce susceptibility to infection and/or replication by MERS-CoV.

The idea that genetic variation at the DPP4 locus may contribute to MERS-CoV susceptibility and infection outcomes is supported by literature. The DPP4 gene contains multiple polymorphisms, some of which have been investigated for their potential effects on MERS-CoV binding/attachment or other aspects of MERS transmission. Kleine-Weber et al. identified 9 polymorphic sites in the DPP4 gene, in regions that encode residues that interact with the MERS-CoV spike protein (29). Several of these polymorphisms appeared to influence MERS-CoV entry and infection in biochemical and virological assays. Similarly, Abbad and colleagues used *in silico* modeling to predict the effects of several polymorphisms in the MERS-CoV interacting region of DPP4 (30). The prevalence of these polymorphisms in Middle Eastern populations, where the MERS outbreak had its greatest impact, is unknown. Recently, Abuelizz et al. (31) reported a number of SNPs in the vicinity of DPP4 in a Saudi Arabian population, some of which have the potential to impart functional consequences for DPP4 by altering expression levels or protein function.

Increased DPP4 abundance was associated with greater viral shedding from the apical surface of infected epithelia early in infection, and peak titers were reached 1 day earlier in the Above-Mean DPP4 group than in the Below-Mean group (Figure 4). Both observations suggest that, generally, greater expression of the viral receptor led to more infection and allowed for more efficient early viral replication. Viral attachment to cells depends on numerous factors, such as access to viral binding protein(s) as well as receptor density, number, and affinity (32). It is possible that the HAE donors in the Above-Mean group represent individuals with increased proportions of DPP4-positive cells. In this setting, individuals with higher receptor expression would have a greater pool of cells susceptible to MERS-CoV infection and support more efficient viral replication and spread. Alternatively, it may be that inter-individual differences in DPP4 abundance reflect differences in the cell surface density of the receptor, which could potentially influence viral binding and entry. Additional immunofluorescence and flow cytometry studies will be helpful in determining to what degree each of these scenarios contribute to the observed donor-dependent difference in viral replication kinetics for MERS-CoV.

We also explored how expression of DPP4 intersects with tropism and spread of MERS-CoV through the airway epithelium. We noted occasional infected ciliated cells during the early rounds of infection. Later, the virus predominantly infected non-ciliated cells. The infection of ciliated cells, despite the fact that ciliated cells represent only a small proportion of the DPP4-positive cell population, highlights the possibility of additional factors that can promote MERS-CoV binding and entry. We note that mRNA for transmembrane protease serine 2 (TMPRSS2), the cell surface serine protease implicated in cleavage/activation of the MERS-CoV spike protein during viral entry into airway epithelia, is abundant in both ciliated and non-ciliated airway epithelia (17, 18, 20). Li et al. recently demonstrated that in addition to DPP4, MERS-CoV S protein binds sialic acid with a preference for α2, 3-linked over α2, 6-linked sialic acid moieties (33). This sialic acid binding specificity could be relevant to MERS-CoV tissue tropism and virus transmission, as α2, 3-linked sialic acids are more commonly found on ciliated cells in the airways (while α2, 6-linked sialic acids predominate on non-ciliated cells) (34–36). It has also been reported that removal of cell surface sialic acid blocks MERS-CoV entry into Calu-3 cells (33), further indicating that sialic acid may be a MERS-CoV co-receptor. Additional experiments will be needed to determine whether sialic acid binding contributes to MERS-CoV tropism in well-differentiated primary cultures of airway epithelia.

The dynamic changes in infected cell types that we observed over the course of MERS-CoV infection suggested that ciliated cells were lost over time (Figure 1), and indeed we found evidence that MERS-CoV triggered shedding of infected cells as infection progressed (Figure 4). Injury and loss of ciliated cells was reported in mice, rhesus macaques, and dromedaries following acute MERS-CoV infection (37–39), and in an autopsy report of fatal MERS (40). Significant shedding of ciliated cells has also been observed in human airway epithelia infected with SARS-CoV (41) and SARS-CoV-2 (42), suggesting that this phenomenon may be a general feature of infection with the more pathogenic coronaviruses. Loss of ciliated epithelia is likely to reduce the integrity of the epithelial barrier and can potentially impair mucociliary clearance in the airways (23). Furthermore, apoptotic or necrotic infected cells may promote antigen presentation and phagocytosis (43). Usually apoptotic cells are cleared by scavenger phagocytes and this process is anti-inflammatory (43). However, in some cases when cells are not cleared by phagocytosis and the apoptotic process is completed, apoptotic cells undergo autolysis and release inflammatory molecules through secondary necrosis which may further exacerbate inflammation and lung injury (44). In our study, cell shedding was greater in epithelia from donors with above average levels of DPP4, indicating that receptor abundance may play a role in determining the severity of this aspect of MERS-CoV pathogenesis in different individuals.

Variability in DPP4 abundance also appeared to have implications for host antiviral responses to MERS-CoV infection. We found that while viral titers had peaked by day 3 or 4 post infection (Figure 3), induction of the interferon response seemed to be suppressed early in the timecourse (Figure 5). Significant upregulation of IFN-β and IFN-λ was seen only at the later timepoints post infection (days 3–5), primarily in the HAE donors with above average DPP4 abundance. The relative timing of interferon production and peak virus titers are critical determinants of virus infection outcomes. In animal models of infection, the administration of type I IFN after maximal replication of SARS-CoV or MERS-CoV increased immunopathology and resulted in lethal infection (45, 46). Thus, the delayed increase in type I IFN expression we observed in HAE at 5 days post infection may contribute to disease pathology in humans, particularly for individuals with relatively high levels of DPP4.

MERS-CoV infection also induced expression of a subset of host innate immune genes, whose upregulation late in the timecourse paralleled the delayed expression of interferons. Most striking was the finding that MERS-CoV infection potently upregulated expression of IP-10/CXCL10. Induction of IP-10/CXCL10 is a critical event in the initiation of immune-mediated acute lung injury and lymphocyte apoptosis. Elevated IP-10/CXCL10 expression has been associated with severe outcomes in both SARS-CoV and MERS-CoV infection (47–50), and patients who develop ARDS following SARS-CoV or H5N1 avian influenza virus infection exhibit high levels of IP-10/CXCL10 in the lungs (47, 51). In keeping with this, studies in IP-10/CXCL10-deficient mice demonstrate that the absence of IP-10/CXCL10 reduces disease severity and improves survival in nonviral and viral ARDS models, suggesting that IP-10/CXCL10-CXCR3 signaling worsens ARDS pathology (52). Based on these observations, we speculate that individual differences in epithelial cell DPP4 expression may impact disease outcomes, in particular by influencing IP-10/CXCL10-induced immune cell infiltration.

In summary, we investigated individual variation in DPP4 expression in well-differentiated primary human airway epithelia. Our results revealed that cells from different airway epithelial donors express variable levels of DPP4 protein, and that this variability translates to measurable differences in viral replication and pathogenesis as well as host innate immune responses during infection. These findings indicate that inter-individual variation in DPP4 abundance is a host factor that partly determines the outcome of MERS-CoV infection, and that individuals with higher DPP4 expression may be at increased risk of developing severe infections. An improved understanding of MERS-CoV infection in human airway epithelia may aid in identifying high risk populations and in designing strategies to reduce viral entry or replication by this pathogenic coronavirus.

## Data Availability Statement

The raw data supporting the conclusions of this article will be made available by the authors, without undue reservation.

## Ethics Statement

The study was approved by the Institutional Review Board at the University of Iowa. Written informed consent to participate in this study was provided by the participants or their legal guardian/next of kin.

## Author Contributions

KL and PM conceived and designed experiments. KL and CW-L performed the experiments. KL and JB performed data analysis and figure preparation and wrote the first draft of the manuscript. PM and JB edited and revised the manuscript. PM provided funding, resources, and supervision. All authors read and approved the submitted version of the manuscript.

## Funding

This work was supported by the National Institutes of Health P01 AI060699.

## Conflict of Interest

The authors declare that the research was conducted in the absence of any commercial or financial relationships that could be construed as a potential conflict of interest.

## Publisher's Note

All claims expressed in this article are solely those of the authors and do not necessarily represent those of their affiliated organizations, or those of the publisher, the editors and the reviewers. Any product that may be evaluated in this article, or claim that may be made by its manufacturer, is not guaranteed or endorsed by the publisher.
